# Health-related quality of life and physical functioning in people living with HIV/AIDS: a case–control design

**DOI:** 10.1186/1477-7525-11-106

**Published:** 2013-06-26

**Authors:** Chidozie Emmanuel Mbada, Olaniyi Onayemi, Yewande Ogunmoyole, Olubusola Esther Johnson, Christopher O Akosile

**Affiliations:** 1 Department of Medical Rehabilitation, College of Health Sciences, Obafemi Awolowo University, Ile – Ife, Nigeria; 2 Department of Dermatology and Veneriology, Faculty of Clinical Sciences, College of Health Sciences, Obafemi Awolowo University, Ile – Ife, Nigeria; 3 Department of Physiotherapy, Obafemi Awolowo University Teaching Hospitals Complex, Ile – Ife, Nigeria; 4 Department of Medical Rehabilitation, College of Health Sciences, Nnamdi Azikiwe University, Nnewi Campus, Nnewi, Anambra State, Nigeria

**Keywords:** Functional exercise capacity, HIV/AIDS, 6MWD, SF-12

## Abstract

**Background:**

Health-Related Quality of Life (HRQoL) and functional exercise capacity are important area of therapeutic interventions needed to improve the general health of People Living with HIV/AIDS (PLWH). However, the relationship between self-report and Performance-based Measure of Functional Capacity (PMFC) of PLWH is still obscure. This study compared the HRQoL and PMFC between a homogenous sample of clinical stage I PLWH and apparently healthy controls.

**Methods:**

This case–control study involved 74 consenting participants (37 PLWH and 37 controls) who completed the self-report SF-12 questionnaire and PMFC assessment using Six Minute Walk Test (6MWT). PMFC was expressed in terms of Six-Minute Walk Distance (6MWD), Six-Minute Walk Work (6MWW) and Maximum oxygen uptake (VO_2_max). Data were analyzed using descriptive statistics of mean and inferential statistics of independent *t*-test, ANOVA and Pearson’s product moment correlation. Alpha level was set at 0.05.

**Results:**

There was no significant difference in the SF-12 Physical-health Component Score (PCS) of PLWH and the controls (p = 0.782). However, the SF-12 Mental-health Component Score (MCS) of PLWH was higher than that of controls (p = 0.040). 6MWD, 6MWW and VO_2_max were significantly lower for PLWH (p < 0.05). Among PLWH, there was no significant gender differences in the PMFC (p > 0.05) while PCS was higher among females. There was no significant correlation between PMFC variables and each of PCS and MCS for PLWH and controls (p > 0.05) respectively.

**Conclusion:**

Self-report physical health of clinical stage 1 PLWH and controls was comparable, while self-report mental health capacity was higher in PLWH than the controls. PMFC of PLWH was significantly lower compared to healthy controls without gender bias. Overall, self-report and performance-based measure of physical functional capacity of PLWH was not inter-related. Therefore understanding the factors that may influence exercise capacity of PLWH may help to develop effective exercise programmes for PLWH.

## Background

The Human Immune-deficiency Virus/Acquired Immunodeficiency Syndrome (HIV/AIDS) epidemic is on the increase around the world with about 33.2 million people living with the disease worldwide [1,2]. Africa is the most affected region with 1.8 – 1.9 million people living with HIV [3]. As at 2003, Nigeria was estimated to have the third highest number of people living with HIV/AIDS in the world, after South Africa and India [4]. There are varying reports on the prevalence rate of HIV/AIDS in Nigeria in the recent years; however, all the reports suggest an upward trend in the prevalence [3,5,6]. The WHO estimates that 3.1% of adults aged 15–49 years are HIV positive in Nigeria [3].

HIV/AIDS is a life-long disease that poses severe threats to the health of millions ensuing in morbidity and mortality around the world [7] and it maintains an insolent stance against interventions as no cure or vaccine has been found for it [7-9]. However, there are praiseworthy advances in medical and pharmacological management of patients with HIV/AIDS with reported successes but not without attendant costs and adverse side effects [10,11]. Complementarily, therapeutic exercise is one of the approaches being explored to help deal with the complications and symptoms of HIV/AIDS [12]. The HIV infects cells of the immune system, destroying or impairing their function leading to progressive deterioration of the immune system called "immune deficiency" [3]. Presentations associated with chronic HIV infection that eventually lead to disability and mortality includes muscle wasting, muscle weakness, fatigue, impaired functional work capacity, depression and decreased quality of life [7].

Various reports have shown that the presence of HIV/AIDS as well as the symptoms and complications associated with HIV/AIDS have a negative effect on the quality of life of People Living with HIV/AIDS (PLWH) [6-9]. Health-Related Quality of Life (HRQoL) refers to quality of life in a clinical setting that includes those dimensions such as global health perceptions, symptom status, functional status, biologic and physiologic variables, individual and environmental characteristics directly affected by the overall state of health [13,14]. Research on health-related outcomes such as HRQoL and functional exercise capacity of PLWH are acclaimed to be significant areas for therapeutic intervention needed to improve the general health of PLWH [15,16].

Functional exercise capacity is believed to be depressed in PLWH [17-19]. The mechanism explaining the relationship between HIV/AIDS and functional exercise capacity is somewhat intricate and complex. Meanwhile, the pathophysiology of the decreased functional exercise capacity has been associated with the effects of HIV/AIDS on anaerobic metabolism, muscle fatigue, and eventual muscle wasting in PLWH [20-23]. Similarly, the immune system of the PLWH and several immunological variables including HIV-1 ribonucleic acid viral load and CD4 cell count has been implicated for decreased functional exercise capacity in PLWH [24,25]. Furthermore, there are available reports suggesting that psychological health such as quality of life decreases as HIV/AIDS progresses, and this may affect the exercise capacity of PLWH [16,20-22,26].

An assessment of functional exercise capacity and the factors that may influence the outcome of exercise in PLWH are required in designing an exercise program. The Six Minute Walk Test (6MWT) has been accepted as one of the important performance-based tool in the assessment of functional exercise capacity and it has been used extensively in various populations including HIV/AIDS [27,28]. In the same vein, patient’s self-reported functional capacity assessed using questionnaires based on indicators of functioning on different activities of daily living have been reported in some studies as valid [29-32]. However, comparison of performance-based and self-rated functional capacity has been reported in literature but with variable results [32-34]. In addition, discrepancies exist in literature as to what extent self-reporting can replace performance based testing [35]. However, there is an apparent dearth of studies comparing self-report and Performance-based Measure of Functional Capacity (PMFC) of PLWH. This study compared the HRQoL and PMFC between a homogenous sample of clinical stage I PLWH and apparently healthy controls.

## Methods

A total of 74 participants (37 PLWH (15 (40.5%) males and 22 (59.5%) females) and 37 age and sex matched apparently healthy controls) volunteered for the study. Consenting PLWH in this study were recruited from the Virology Research Clinic (VRC) of Obafemi Awolowo University Teaching Hospitals Complex (OAUTHC) Ile-Ife, Nigeria. In order to ensure homogeneity of sample, PLWH in clinical stage I of the disease who were 18 years and older and were recruited. Clinical stage I of HIV/AIDS refers to asymptomatic/acute HIV infection, with persistent generalized lymphadenopathy [36,37]. All the PLWH volunteers were on anti-retroviral therapy (HAART). Consenting participants that had a history of unstable angina and myocardial infarction during the previous month and those that were already involved in an exercise program were also excluded. The apparently controls in this study included patients’ relatives and health workers at the VRC. The ethical approval for this study was obtained from the ethical committee of the OAUTHC. The VRC gave permission for the study to be carried out at the centre. Each participant gave informed consent to participate in the study. The participants’ ages were recorded while weight and height were also measured and recorded.

Health-Related Quality of Life (HRQoL) was assessed using the Medical Outcomes Study Short-Form 12 questionnaire (SF-12). As a generic instrument, the SF-12 has been shown to be valid in both apparently healthy individuals and PLWH [38-40]. The SF-12 is a 12-item version of the Medical outcomes study Short Form-36 (SF-36) addressing physical functioning, role limitations due to physical health, role limitations due to emotional health, social functioning, bodily pain, general health perceptions, vitality, and mental health [40-42]. The SF-12 reduces item redundancy and the burden of data requirements for both investigators and patients [41]. The SF-12 yields two summary scores; the Physical-health Component Score (PCS) and the Mental-health Component Score (MCS). The participants filled the questionnaire between 2–3 minutes while sitting comfortably on a chair. The PCS of the SF-12 was used in this study as a measure of self-report functional capacity.

Performance-based Measure of Functional Capacity (PMFC) was assessed using the 6-minute walk test (6MWT) in accordance with the American Thoracic Society [43]. This test measures the distance that a participant can quickly walk on a flat, hard surface in a period of 6 minutes referred to as the 6-minute walk distance (6MWD). A distance of 30 m was marked on the hallway with white chalk. The participants were then asked to walk back and forth in the hallway for six minute from one end of the 30 m distance mark to another, pivoting briskly at each end and continue walking without hesitation. Participants that became exhausted were permitted to slow down, to stop, and to rest as necessary. They were allowed to lean against the wall while resting, but resume walking as soon as they were able to. If the participant stopped before the 6 minutes were up and refused to continue, a chair was taken to the participant to sit on, and the walk discontinued. When the test was completed, the spot where the participant stopped was marked and the total distance walked (i.e. 6MWD) in meters was recorded. No encouragement was given during the performance of the test. Data from participants that fail to complete the test were excluded from analysis. PMFC was expressed in terms of Six-Minute Walk Distance (6MWD), Six-Minute Walk Work (6MWW) and Maximum oxygen uptake (VO_2_max).

## Computations

I.

$$\begin{array}{l} \mathrm{Six}\;\mathrm{Minute}\;\mathrm{Walk}\;\mathrm{Work}\;\left(6\mathrm{MWW}\right)\;\left(\mathrm{kg}/m\right)\;\\ \;=\;6\mathrm{MWD}\;\times \;\mathrm{body}\;\mathrm{weight} \end{array}$$

[44].

II.

$$\begin{array}{l} \mathrm{Maximum}\;\mathrm{oxygen}\;\mathrm{uptake}\;\left({\mathrm{VO}}_{2}\max\right)\;\left(\mathrm{mL}/\mathrm{kg}/\mathrm{minute}\right)\\ \;=\;\left(\mathrm{speed}\left(\mathrm{mmin}-1\right)X\;0.1\right)\;+\;3.5 \end{array}$$

[45].

### Statistical analysis

Statistical Package for Social Sciences (SPSS) software (version 16) was used for the data analysis. Data were analyzed using descriptive statistics of mean and standard deviation. Inferential statistics of independent *t*-test was used to compare variables between the PLWH and their healthy controls. One-Way ANOVA and LSD post-hoc comparison was used to compare variables between groups and by gender. Pearson’s product moment correlation analysis was used to test the relationships between variables. Alpha level was set at p < 0.05.

## Results and discussion

### Result

The participants’ ages ranged between 20 and 54 years. The physical characteristics of the participants are presented in Table 1. The PLWH and their healthy controls were comparable in age, weight, height and body mass index (p > 0.05).

**Table 1:** **Independent *t*-test comparison of the general characteristics of PLWH and control group**

	**PLWH**	**Control group**		
**Variable**	**X±SD**	**X±SD**	**t - cal**	**p-value**
Age	35.68 ± 7.71	35.73 ± 7.88	−0.30	0.976
HT	1.67 ± 0.79	1.66 ± 0.08	0.344	0.739
WT	63.49 ± 12.25	67.12 ± 11.35	−1.324	0.190
BMI	22.77 ± 4.17	24.31 ± 4.24	−1.583	0.118

Abbreviations: *HT* Height, *WT* Weight, *BMI* Body mass index.

The independent *t*-test comparison of the PCS and MCS between the PLWH and control group are shown in Table 2. The control group had a higher PCS than the PLWH (51.92 ± 7.35 vs. 52.40 ± 7.61) but was not statistically significant (p > 0.05). The MCS of the PLWH was found to be significantly higher than the MCS of the control group (p = 0.04). Table 3 shows the independent *t*-test comparison of performance-based measures of functional capacity between the PLWH and control group. From the result, the control group had significantly higher 6MWD, 6MWW and VO_2_max respectively (p = 0.001).

**Table 2:** **The independent *t *test comparison of the PCS and MCS of PLWH and control group**

	**PLWH**	**Control group**		
**Variable**	**X±SD**	**X±SD**	**t-cal**	**p-value**
PCS	51.92 ± 7.35	52.40 ± 7.61	−0.277	0.782
MCS	73.00 ± 13.00	65.26 ± 9.27	2.952	0.040*

* Indicates significant difference between groups.

Abbreviations: *PCS* Physical-health Component Score, *MCS* Mental-health Component Score.

**Table 3:** **Independent *t*-test comparison of the measures of functional exercise capacity between PLWH and control group**

	**PLWH**	**Control group**		
**Variable**	**X±SD**	**X±SD**	**t - cal**	**p-value**
6MWD	442.95 ± 93.32	556.07 ± 93.41	−5.211	0.001*
6MWW	27853 ± 7501.63	37056 ± 7433.32	−5.301	0.001*
VO_2_max	30.07 ± 5.60	36.86 ± 5.60	−5.211	0.001*

* Indicates significant difference between groups.

Abbreviations: *6MWD* 6 minute walk distance, *6MWW* 6 minute walk work, *V0*_*2*_*max* Peak oxygen uptake.

One-Way ANOVA and LSD post-hoc comparison of the performance-based functional exercise capacity measures, PCS and MCS by gender is presented in Table 4. LSD post-hoc test was used to elucidate where the differences observed in the F-ratio lies. From the result, there were no significant gender differences in 6MWD, 6MWW and VO_2_max among PLWH (p > 0.05). However, female participants with HIV/AIDS had significantly higher PCS (48.97 ± 8.33 vs. 53.92 ± 5.99; p < 0.05) while MCS was comparable (p > 0.05). On the other hand, the male controls had significantly higher values for 6MWD, 6MWW and VO_2_max (p < 0.05) respectively, while PCS and MCS were comparable between both gender (p > 0.05). Pearson product moment correlation between functional exercise capacity variables (6MWD, 6MWW and VO_2_max) and each of PCS and MCS is shown in Table 5. From the result, there was no significant correlation between PCS and the performance-based functional capacity measures for the PLWH and control groups respectively (p > 0.05). Also, there was no significant correlation between MCS and the performance-based functional capacity for the PLWH and control groups respectively (p > 0.05).

**Table 4:** One-Way ANOVA and LSD post-hoc comparison of performance-based functional exercise capacity measures, PCS and MCS by gender

	**PLWH**		**Control group**			
	**Male**	**Female**	**Male**	**Female**		
	**n = 15**	**n = 22**	**n = 15**	**n = 22**		
**Variable**	**Mean ± S.D**	**Mean ± S.D**	**Mean± S.D**	**Mean ± S.D**	**F-ratio**	**p-value**
6MWD	466.22 ± 89.9^a^	427.09 ± 94.32^a^	600.09 ± 112.59^b^	526.07 ± 64.65^c^	12.356	0.001
6MWW	29001 ± 5648.5^a^	27070 ± 8679.91^a^	41594 ± 8149.44^b^	33962 ± 5070.20^c^	14.287	0.001
VO_2_max	2903.6 ± 564.85^a^	2710.5 ± 857.99^a^	4162.9 ± 814.94^b^	3399.7 ± 507.01^c^	14.287	0.001
PCS	48.97 ± 8.33^a^	53.92 ± 5.99^b^	53.74 ± 5.77	51.49 ± 8.66	1.662	0.183
MCS	73.62 ± 9.74^a^	72.58 ± 15.03^a^	64.03 ± 7.50^b^	66.09 ± 10.39^b^	2.960	0.038

Abbreviations: *6MWD* 6 minute walk distance, *6MWW* 6 minute walk work, *VO*_*2*_*max* Peak oxygen uptake, *PCS* Physical-health Component Score, *MCS* Mental-health Component Score, *Superscripts (*^*a,b,c,d*^*).*

For a particular variable, mode means with different superscript are significantly (P<0.05) different. Mode means with same superscripts are not significantly (P>0.05) different. When only one contrast is significant, one of the cell means has no superscript attached. The pair of cell means that is significant has different superscripts.

**Table 5:** Pearson product moment correlation between functional exercise capacity variables and each of PCS and MCS

	**PLWH**	**Control group**
**Variable**	**r (p-value)**	**r (p-value)**
**PCS**		
6MWD	0.120 (0.479)	0.186 (0.271)
6MWW	0.007 (0.966)	−0.087 (0.607)
VO_2_max	0.117 (0.485)	0.186 (0.271)
**MCS**		
6MWD	0.006 (0.973)	−0.176 (0.296)
6MWW	0.168 (0.321)	0.015 (0.932)
VO_2_max	−0.016 (0.926)	−0.176 (0.296)

Abbreviations: *PCS* Physical-health Component Score, *MCS* Mental-health Component Score, *6MWD* Six minute walk distance, *6MWW* 6 minute walk work, *V0*_*2*_*max* Peak oxygen uptake.

## Discussion

This study assessed the HRQoL and PMFC of a homogenous sample of clinical stage I PLWH and their age-and-sex matched healthy controls. The PCS of the SF-12 was employed in this study as a self-report measure of physical functional capacity. From the result, there was no significant difference in the PCS between PLWH and the controls. The finding on comparable PCS between the PLWH and the controls may be a reflection of the clinical stage of the PLWH. Several studies using the SF-12 have shown that physical and mental capacity decreases as the severity of HIV/AIDS increases [46-49]. This present study recruited a homogenous sample of PLWH in clinical stage 1 [asymptomatic stage] who were currently on anti-retroviral therapy [HAART]. The symptoms and complications of HIV/AIDS including muscle wasting, fatigue and disability as well as prevalence of opportunistic infections have been shown to be significantly reduced with the introduction of highly active HAART [46,50]. It is adduced that the asymptomatic stage and the effect of the HAART in retarding the progression of HIV/AIDS disease may have led to the comparable self-reported physical capacity between PLWH and the controls. Furthermore, the comparable PCS among the groups may be due to the young age of the participants. In a cross sectional study, Nojomi et al. [48] found PLWH below age 35 years to have significantly better PCS than other age groups. The mean age (35.68 ± 7.71 years) of PLWH that participated in this study is similar to that reported by Nojomi et al. [48] as having better physical capacity than older PLWH.

The result of this study showed that the self-reported MCS of PLWH was significantly higher than the healthy controls. This finding contradicts the results of previous studies that have found low mental health in PLWH [51-54]. However, similar to the finding of this study, Ostrow et al. [55] found that HIV/AIDS had a negative effect on the physical health of PLWH, but not on their mental health and attributed finding to the positive effect of HAART on the mental functioning of PLWH. HAART has been reported to have a positive effect on the life expectancy of PLWH, changing the view of HIV/AIDS from a deadly disease to a potentially manageable disease and consequently lead to improved survival and reduction in opportunistic infections [47]. Apart from the stage of no/minimal presence of symptoms of HIV/AIDS, accessibility to HAART by PLWH at the clinic where this study was conducted may have led to improvement in the PLWH perspective of the disease resulting in a reported better mental health capacity. In addition, the trend of people with terminal illnesses such as HIV/AIDS towards a more spiritual disposition may also explain the significantly higher mental health capacity of PLWH found in this study. Nigeria is a religious country where nearly everyone belongs to one religion or the other. Anecdotal and empirical reports indicated that Nigerian patients with chronic illnesses have high level of religious inclinations [56,57]. High levels of religious inclinations have been reported among patients with HIV/AIDS [58-62]. The possible religious disposition portends that PLWH may translate into being positive-minded and over-psyched. PLWH often adopt spirituality as a coping strategy to help reframe their lives and also to bring a sense of meaning and purpose to their lives in the face of an often devastating situation [63-65]. Some other studies have found spirituality to be positively associated with improvements in life satisfaction, functional health status, health-related quality of life, and overall well-being in PLWH [57,60,66,67]. Anecdotally, most of the patients seen in the course of this study often speak in terms of their anticipation for better health rather than accepting the present realities of the HIV/AIDS infection. This form of communication may account for the higher mental health component in PLWH compared with the controls.

From this study, performance-based functional capacity [6MWD, 6MWW and VO_2_ max] of PLWH was found to be significantly lower than that of the controls. The result on lower functional capacity among PLWH is consistent with previous reports [68-70]. Specifically, from the result of this study, 6MWD by the PLWH was significantly shorter than that of the controls. This may be attributed to muscle wasting and weakness, increased muscular fatigability, impaired psychosocial and functional capacity that has been associated with HIV infection [7]. The work done during the 6MWT as revealed by the 6MWW in this study was also found to be significantly lower among the PLWH. 6MWW is a product of the 6MWD [in meters] and total body weight [in kilograms] [44]. Dourado [68] suggested that several factors including demographic, anthropometric and physiological factors have an effect on the result of the 6MWT. Obese participants usually have reduced lean body mass, leading to a shorter 6MWD. Carter et al. [71] suggested that the weight of the participant may have an effect on the amount of energy used up during the work and thus the distance ambulated. It is believed that this may improve the accuracy and extend the utility of the 6MWT [71]. Therefore, the 6MWW is a better reflector of the work done during 6MWT than the 6MWD. Few studies among healthy subjects and patients with chronic obstructive pulmonary disease [COPD] have employed the use of the 6MWW [71,72]. However, there seems to be no available study on 6MWW in PLWH.

VO_2_ max of PLWH in this study were found to be lower than that of the healthy controls. This finding is similar to a report by Rogea et al. [73] who found significantly lower working capacity and the trend towards reduced VO_2_ max in PLWH compared to healthy controls. Reduced VO_2_ max in PLWH may be as a result of reduced oxygen uptake and reduced energy expenditure by PLWH. Pothoff et al. [17] found reduced VO_2_ max in PLWH at the anaerobic threshold maximal exercise levels when compared with healthy controls. Stringer et al. [18] suggested physiological deconditioning as a causative factor of reduced VO_2_ max in PLWH. However, physiological deconditioning alone cannot account for the severity of the limitation associated with PLWH [69]. Another explanation for the reduced VO_2_ max observed in PLWH especially those in clinical stage I may be due to reduced ability of the exercising musculature to extract and utilize oxygen and highly active anti-retroviral therapy [HAART] also appears to limit peak oxygen uptake [19].

From this study, there were no significant gender differences in the PMFC. However, females with HIV/AIDS had significantly higher PCS while MCS was comparable between both genders. In contrast, the male control subjects had significantly higher values for 6MWD, 6MWW and VO_2_max respectively, while PCS and MCS were comparable between both genders. To date, there is limited data concerning women with HIV/AIDS as compared to available data on their male counterparts. Furthermore, there seems to be no available studies comparing functional exercise capacity between male and female PLWH. On the other hand, in concert with the findings of this study, Holzemer et al. [74] demonstrated that HIV positive women possessed higher self-reported quality of life than infected men. Also, O’Keefe and Wood [75] in a study conducted in South Africa found that black females with HIV had higher PCS compared with men with HIV.

From this study, there was no significant correlation between self-report and performance-based measure of functional capacity of PLWH and the healthy controls. Literature on the correlation between self-report and performance-based measure of functional capacity of PLWH is scarce. However, a study by Vidrine et al. [76] revealed no association between the functional status and physical and mental health components assessed with the SF-12 in PLWH.

### Clinical implications of the study

This study confirms the findings from many investigators that PLWH are severely deconditioned and face functional aerobic impairment compared to the general population. However, to our knowledge, there seems to be a dearth of studies that have actually compared PLWH with healthy controls. This study found that those with HIV/AIDS had significantly lower functional exercise capacity compared to healthy persons of the same age and gender. In part, the observed significantly lower VO_2_ max could have resulted from impaired physical fitness revealed by significant lower performance in 6MWT among PLWH. Laura et al. [77] found 6MWT as a valid locomotor performance test of functional capacity in PLWH. Some studies have implicated the use of HAART among PLWH in decreased locomotor performance as a result of direct adverse effects on the muscle and peripheral neurotoxicity [50,78]. Furthermore, this study found that psychosocial constructs such as HRQoL does not influence functional exercise capacity in clinical stage I PLWH. It is implied that HRQoL and functional exercise capacity performance in PLWH are not inter-dependent. Therefore, therapeutic interventions targeted at improving functional exercise capacity in PLWH may not adequately address psychosocial impairment resulting from HIV/AIDS infection.

### Limitations of the study

The outcome of this study cannot be generalized to PLWH in other clinical stages. This is because the clinical stage of HIV/AIDS as well as the progression of the disease has a negative effect on the self-reported quality of life of PLWH [48]. Furthermore, the healthy controls for this study constituted of patients relatives and some health workers at the centre of the study. Relative and caregivers of PLWH have been linked with depression, psychological burden and stress which may affect their overall mental health as a result of poverty and financial struggles induced by HIV/AIDS [79,80]. Therefore, a selection of healthy controls from populations other than those who have contacts with PLWH may yield a different mental health assessment result. Nonetheless, future studies are needed to put these reasonings into empirical analysis.

## Conclusion

Self-report physical health capacity of PLWH and healthy controls was comparable, while self-report mental health capacity was higher in PLWH than the controls. Performance-based functional capacity was lower in clinical stage 1 PLWH compared to the healthy controls. Overall, self-report and performance-based measure of functional exercise capacity of PLWH was not inter-related. Therefore understanding the factors that may influence exercise capacity of PLWH may help to develop effective exercise programmes for PLWH.

## Competing interests

The authors declare no competing interest.

## Authors’ contributions

CEM conceived the idea for this study, participated in the design of methodology and data collection and analysis and prepared the final manuscript for publication. OO participated in the design of the study's methodology, participated in the interpretation of data and drafted the manuscript. YO participated in the design of methodology and data collection and analysis and drafted the manuscript. OEJ and COA participated in the design of the study's methodology and drafted the manuscript. All authors read and approved the final manuscript.
